# Supplementary material for: Causal Relationship between Aging and Anorexia Nervosa: A White-Matter-Microstructure-Mediated Mendelian Randomization Analysis
Source: Biomedicines. 2024 Aug 16;12(8):1874. doi: 10.3390/biomedicines12081874 (PMC11351342; doi:10.3390/biomedicines12081874)
Supplement: Supplementary file 1 [file biomedicines-12-01874-s001.zip › Supplementary Figure/Figure S4/Singe SNP plot of MR analysis for TL to ubm-b-1572.pdf]

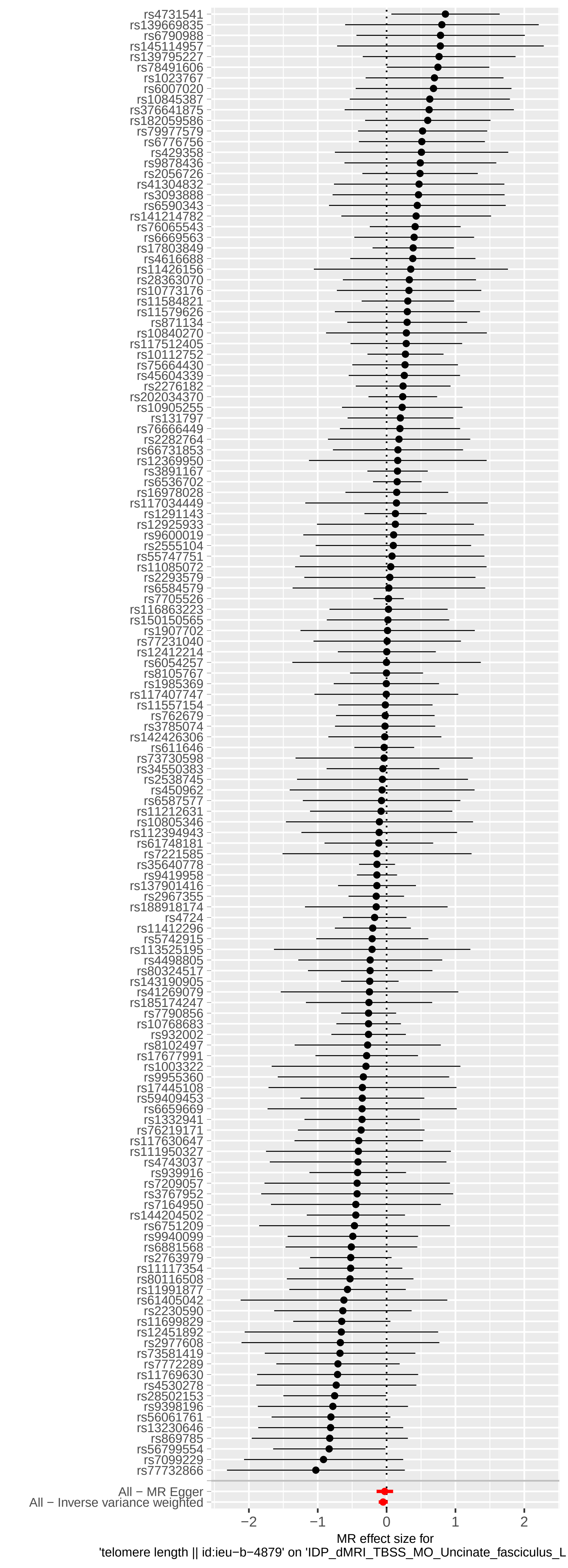
rs4731541
rs139669835
rs6790988
rs145114957
rs139795227
rs78491606
rs1023767
rs6007020
rs10845387
rs376641875
rs182059586
rs79977579
rs6776756
rs429358
rs9878436
rs2056726
rs41304832
rs3093888
rs6590343
rs141214782
rs76065543
rs6669563
rs17803849
rs4616688
rs11426156
rs28363070
rs10773176
rs11584821
rs11579626
rs871134
rs10840270
rs117512405
rs10112752
rs75664430
rs45604339
rs2276182
rs202034370
rs10905255
rs131797
rs76666449
rs2282764
rs66731853
rs12369950
rs3891167
rs6536702
rs16978028
rs117034449
rs1291143
rs12925933
rs9600019
rs2555104
rs55747751
rs11085072
rs2293579
rs6584579
rs7705526
rs116863223
rs150150565
rs1907702
rs77231040
rs12412214
rs6054257
rs8105767
rs1985369
rs117407747
rs11557154
rs762679
rs3785074
rs142426306
rs611646
rs73730598
rs34550383
rs2538745
rs450962
rs6587577
rs11212631
rs10805346
rs112394943
rs61748181
rs7221585
rs35640778
rs9419958
rs137901416
rs2967355
rs188918174
rs4724
rs11412296
rs5742915
rs113525195
rs4498805
rs80324517
rs143190905
rs41269079
rs185174247
rs7790856
rs10768683
rs932002
rs8102497
rs17677991
rs1003322
rs9955360
rs17445108
rs59409453
rs6659669
rs1332941
rs76219171
rs117630647
rs111950327
rs4743037
rs939916
rs7209057
rs3767952
rs7164950
rs144204502
rs6751209
rs9940099
rs6881568
rs2763979
rs11117354
rs80116508
rs11991877
rs61405042
rs2230590
rs11699829
rs12451892
rs2977608
rs73581419
rs7772289
rs11769630
rs4530278
rs28502153
rs9398196
rs56061761
rs13230646
rs869785
rs56799554
rs7099229
rs77732866
All − MR Egger
All − Inverse variance weighted
−2
−1
0
1
2
MR effect size for
'telomere length || id:ieu−b−4879' on 'IDP_dMRI_TBSS_MO_Uncinate_fasciculus_L
